# Deep multimodal biomechanical analysis for lower back pain rehabilitation to improve patients stability

**DOI:** 10.3389/fbioe.2025.1631910

**Published:** 2025-11-07

**Authors:** Muhammad Abrar Ashraf, Yanfeng Wu, Shaheryar Najam, Mohammed Alshehri, Yahya AlQahtani, Hanan Aljuaid, Ahmad Jalal, Hui Liu

**Affiliations:** 1 Guodian Nanjing Automation Co., Ltd, Nanjing, China; 2 Department of Electrical and Computer Engineering Riphah International University, Islamabad, Pakistan; 3 Department of Electrical Engineering, Bahria University, Islamabad, Pakistan; 4 Department of Computer Science, King Khalid University, Abha, Saudi Arabia; 5 Department of Informatics and Computer Systems, King Khalid University, Abha, Saudi Arabia; 6 Computer Sciences Department, College of Computer and Information Sciences, Princess Nourah bint Abdulrahman University (PNU), Riyadh, Saudi Arabia; 7 Faculty of Computer Science, Air University, Islamabad, Pakistan; 8 Department of Computer Science and Engineering, College of Informatics, Korea University, Seoul, Republic of Korea; 9 School of Future Technology, Nanjing University of Information Science and Technology, Nanjing, China; 10 Cognitive Systems Lab, University of Bremen, Bremen, Germany

**Keywords:** rehabilitation, data acquisition, depth sensing, biomechanical analysis, machine learning, intention recognition, healthcare system

## Abstract

**Introduction:**

Advancements in artificial intelligence are transforming rehabilitation by enabling scalable, patient-centric solutions within modern healthcare systems. This study introduces 3D-PoseFormer, a deep multimodal framework designed for the telerehabilitation of individuals with lower back pain (LBP).

**Methods:**

The proposed system performs automated data acquisition using synchronized RGB and depth video streams to enable real-time, markerless, and sensor-free analysis of physiotherapy exercises. From the depth sensing module, 3D body joint positions are extracted and used to generate SMPL-based mesh vertices for detailed biomechanical analysis and postural representation. Simultaneously, RGB frames are processed using keypoint detection algorithms—Shi-Tomasi, AKAZE, BRISK, SIFT, and Harris corner detection. Extracted features are enhanced through semantic contour analysis of segmented body parts to capture localized appearance-based information relevant to LBP therapy. The fused multimodal features are then passed to a Transformer-based machine learning model that captures temporal motion patterns for accurate exercise classification and human intention recognition.

**Results:**

The system removes the need for wearable sensors and supports autonomous, continuous monitoring in home-based rehabilitation. Validation on the KIMORE dataset (baseline, including rehabilitation exercises by patients with lower back pain), mRI dataset (rehabilitation exercises), and UTKinect-Action3D dataset (comprising diverse subjects and activity scenarios) achieved state-of-the-art accuracies of 94.73%, 91%, and 94.2%, respectively.

**Discussion:**

Results demonstrate the robustness, generalizability, and clinical potential of 3D-PoseFormer in AI-assisted rehabilitation, offering a scalable and intelligent healthcare system for remote physiotherapy and patient monitoring.

## 1 Introduction

Lower back pain (LBP) is a prevalent musculoskeletal disorder that affects spinal posture, mobility, and quality of life. Rehabilitation for LBP often demands sustained physiotherapy involving repetitive, supervised exercises. However, conventional rehabilitation requires regular clinical visits, posing accessibility barriers for individuals in remote, rural, or resource-constrained settings. Moreover, unsupervised home exercises risk incorrect execution, potentially worsening patient outcomes. Furthermore, recent advancements in artificial intelligence and computer vision have enabled promising alternatives for automated rehabilitation. Yet, most image-based systems struggle with real-world challenges such as occlusion, appearance variation, depth ambiguity, and dependency on camera viewpoints (Wang et al., 2011; Cao et al., 2017). These limitations hinder accurate biomechanical analysis and compromise the reliability of unsupervised assessment in home environments.

Recent rehabilitation research has also emphasized cross-dimensional multimodal assessment, where visual modalities are fused with physiological electrical signals to enhance clinical reliability. For example (Ao et al., 2023), employed sEMG-based muscle synergy analysis for gesture recognition, demonstrating the value of electrophysiological cues. Related studies have shown that combining vision with surface EMG improves motor intention decoding (Zhang et al., 2019) and supports more accurate rehabilitation monitoring (Xia et al., 2020). While these multimodal approaches show promise for enhanced accuracy, they introduce practical challenges including increased hardware complexity, user compliance issues with wearable sensors, and potential discomfort during extended use. Consequently, vision-only systems remain valuable for applications requiring non-invasive deployment, minimal infrastructure requirements, and broader accessibility across diverse user populations and environmental conditions.

To address these challenges, we propose 3D-PoseFormer, a deep multimodal framework tailored for remote lower back pain rehabilitation. The system leverages RGB and depth video streams to enable real-time physiotherapy analysis without wearable sensors or physical markers. From depth images, we extract 3D joint locations and reconstruct full-body meshes using the Skinned Multi-Person Linear (SMPL) model (Loper et al., 2015), capturing precise pose and shape parameters. Concurrently, RGB images are processed via classical keypoint detectors (Shi-Tomasi, AKAZE, BRISK, SIFT, Harris) and enhanced through semantic contour extraction to localize anatomical features. These complementary features are fused into a unified representation. These complementary features are fused into a unified representation and passed to a Transformer-based architecture (Vaswani et al., 2017; Liu et al., 2022). This approach effectively models temporal dynamics for robust exercise classification and correctness evaluation.

The proposed 3D-PoseFormer directly addresses prior limitations by using depth-based 3D joint reconstruction and mesh modelling to handle occlusion and appearance variation, while multimodal RGB-D inputs with Transformer-based temporal modelling mitigate depth ambiguity and viewpoint dependency. These design choices ensure robust rehabilitation analysis in realistic scenarios. The framework integrates structural body modelling, appearance cues, and temporal context without requiring physical markers and complements clinician supervision.

We evaluate our system on three public datasets KIMORE (Capecci et al., 2019), mRI, and UTKinect-Action3D achieving state-of-the-art performance (94.73%, 91.0%, and 94.2%, respectively), thereby demonstrating its generalizability and clinical relevance.

Main contributions of this work are as follows.

- We present a novel rehabilitation framework free from external sensors or physical markers, combining depth-based 3D mesh reconstruction with RGB-based 2D keypoint extraction for accurate biomechanical assessment.
- We propose DKP-Net-24, a novel keypoint extraction framework for robust 3D keypoint estimation from depth-based silhouettes under varying arm poses. It employs specialized image processing pipelines to adapt dynamically to different body alignments, ensuring reliable motion tracking for rehabilitation assessment.
- We propose a unique feature fusion technique that combines 2D and 3D keypoints, integrating 2D appearance features, 3D mesh geometry, and semantic contours into a unified feature vector, coupled with Transformer-based classification.

## 2 Literature review

In the domain of exercise assessment and recognition, a wide variety of technologies have been explored. Inertial Measurement Units (IMUs) are one of the most commonly used tools due to their portability and ability to capture fine-grained motion data. Şahin (2024) reviewed wearable technologies in physiotherapy and rehabilitation, highlighting their applications in monitoring movement, sleep, and managing chronic health conditions. Despite the promising results, the need to wear multiple sensors can reduce practicality and user comfort in non-clinical environments.

Gumaei et al. (2019) proposed a hybrid deep learning model combining SRUs and GRUs for multimodal wearable sensor–based human activity recognition, achieving 99.80% accuracy on the MHEALTH dataset and about 95.70% in 10-fold cross-validation. Chang et al. (2011) used Kinect for posture coaching, achieving 91.9% accuracy in pose classification and 93.75% in trajectory recognition, but faced issues with low light and cluttered backgrounds. Yang et al. (2012) achieved 85% accuracy for gait-based exercise recognition with Kinect, but performance dropped when users were occluded or faced away. Barabas et al. (2019) developed a Kinect-based platform for monitoring elderly movements and detecting falls in indoor settings, achieving approximately 92% accuracy in fall detection.

Recent works have also explored telerehabilitation and intelligent exercise monitoring using multimodal and sensor-based approaches. Ashraf et al. (2025) proposed a telerehabilitation system for elderly healthcare using physical exercise monitoring, while Awan et al. (2024) introduced a robust exercise-based telerehabilitation framework tailored for elderly healthcare services. Tayyab and Jalal (2025) developed a machine learning–based system for disabled rehabilitation monitoring and healthcare recognition. Similarly, Akhter et al. (2023) presented a deep skeleton modeling approach with hybrid hand-crafted cues for exercise recognition. Fatima et al. (2024) designed a feature extraction strategy combining full-body and geometric features for sports interaction recognition, whereas Nadeem et al. (2020) applied multidimensional features and a Markov model for accurate physical activity recognition in smart health fitness. Afsar et al. (2023) employed deep learning models with body-worn sensors for sports activity recognition in exergaming, complemented by studies such as Khan et al. (2025), Javeed and Chelloug (2022), and Kaynat et al. (2025), who applied artificial neural networks, gesture recognition, and dynamic features for immersive fitness and wearable-sensor–based exergaming systems. Tayyab et al. (2025) proposed a hybrid deep learning approach combining key body descriptors for sports activity recognition, while Nazar and Jalal (2025) developed wearable sensor–based activity classification methods for intelligent healthcare monitoring. Furthermore, Kamal et al. (2025) proposed a holistic pose estimation and dynamic motion analysis framework for telerehabilitation of physically disabled individuals, demonstrating the potential of deep models in clinically relevant rehabilitation systems.

More recently, RGB-based approaches have gained attention due to their non-intrusive and sensor less nature. Gupta et al. (Gupta et al., 2020) reviewed various RGB video-based human activity recognition models, where several architectures such as 3D CNNs and LSTMs reached 80%–85% accuracy on different movement datasets. However, the sensitivity of RGB approaches to illumination changes, camera placement, and background noise remains a significant challenge. Li et al. (Li et al., 2021) developed an action recognition system using RGB video and graph convolution networks, achieving 82.4% accuracy, but struggled with frame drops and keypoint inaccuracies under occlusion. Aubry et al. (2019) proposed an action recognition approach using 2D skeletons extracted from RGB videos and CNN-based classification, achieving 83.32% (cross-subject) and 88.78% (cross-view) accuracies on the NTU RGB+D dataset with ResNet. Xu et al. (2021) developed a dual-stream model integrating scene images with human skeleton data for action recognition, achieving 94.10% accuracy on benchmark datasets. However, real-world robustness was limited.

Hamdy et al. (2024) proposed a transformer-based model for classifying rehabilitation exercises, achieving 91.96% accuracy to enhance physical therapy assessment and monitoring. However, performance dropped when joint extraction was inaccurate. Recently, 3D human reconstruction methods, especially those using the SMPL model, have shown promise in exercise assessment, providing a detailed understanding of body movement beyond traditional 2D or depth-based methods. Zanfir et al. (2018) developed a 3D pose estimation pipeline using SMPL-based reconstruction, achieving 87.2% accuracy in fitness activity analysis. Kanazawa et al. (2018) introduced Human Mesh Recovery (HMR), using SMPL for 3D pose estimation from a single RGB image, laying the foundation for marker less 3D exercise assessment. Saqlain et al. (2022) introduced 3DMesh-GAR, a 3D human body mesh-based approach for group activity recognition from RGB frames, achieving 93.6% accuracy on the Collective Activity Dataset.

Kocabas et al. (2020) introduced VIBE, which generates temporally coherent SMPL parameters, achieving 86.3% accuracy in action recognition despite motion blur and occlusions. Pavlakos et al. (2017) used volumetric prediction of 3D meshes for activity recognition, reaching 83.7% accuracy in gesture-based fitness datasets. These studies underline the growing relevance of 3D reconstruction techniques, particularly those involving SMPL, in advancing the field of exercise assessment. By capturing pose and shape in a camera-invariant and rotation-robust format, SMPL opens new avenues for tele-rehabilitation, automated posture correction, and non-intrusive fitness coaching.

## 3 Methodology

**FIGURE 1:**
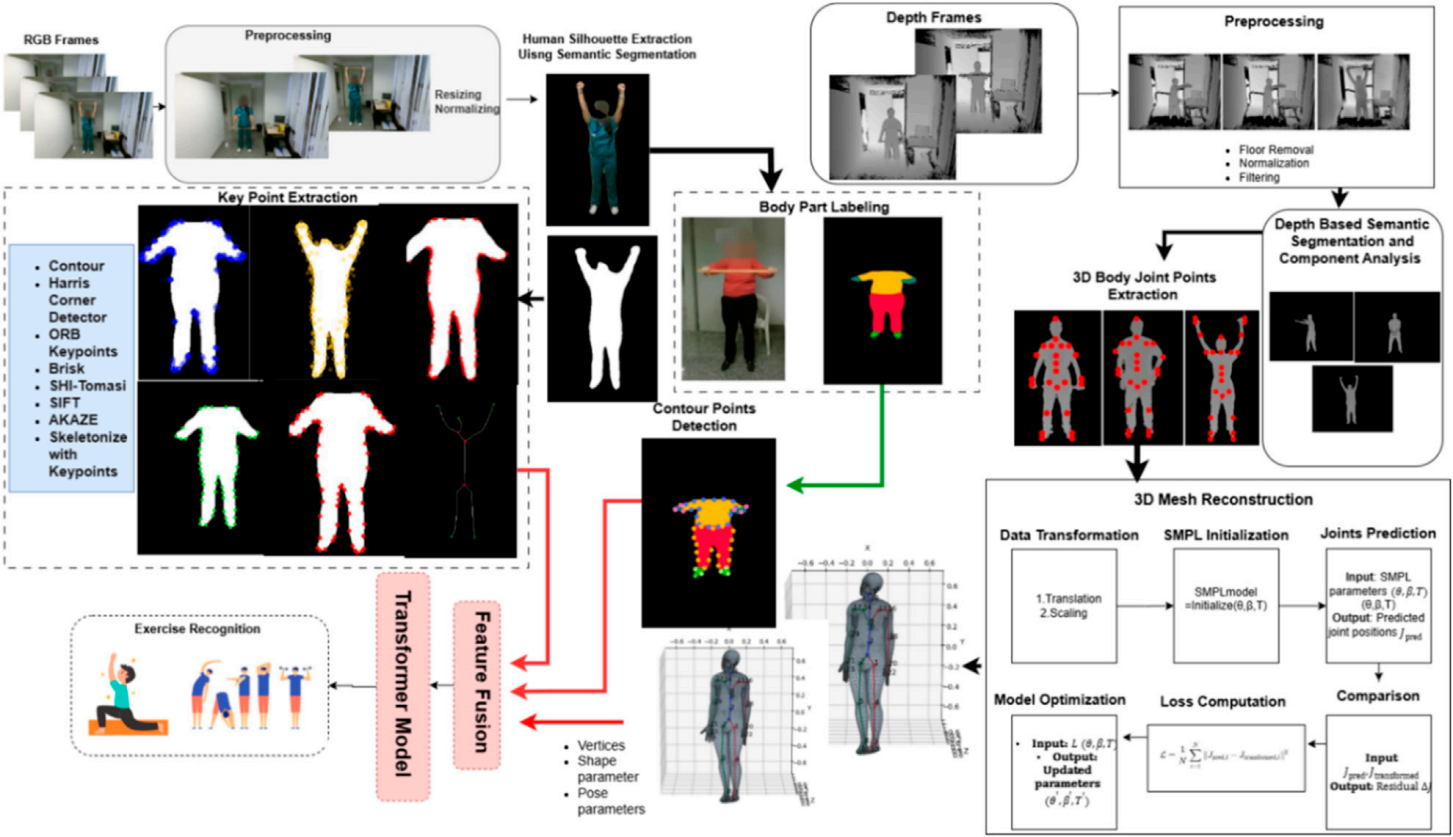
3D-PoseFormer: a deep multimodal pipeline for telerehabilitation of disabled patients via 3D body mesh workflow and part-aware keypoint estimation.

The methodology has two phases: RGB-Keypoint Detection (RGB-KPD), where RGB images were processed to estimate the human pose using keypoints detection algorithms. The second phase is Depth-based Mesh Generation (D-Mesh). In the depth image processing phase, the proposed system extracts human silhouettes and detects 3D body joint positions from depth images. These are then passed to the SMPL model to generate detailed 3D body mesh vertices, along with pose and shape parameters. In the RGB-KPD phase, RGB images are processed to extract complementary visual features. Silhouettes are analyzed using multiple keypoint detection techniques, including Shi-Tomasi, AKAZE, BRISK, SIFT, Harris corner, and contour-based analysis. Body part parsing is performed using a pre-trained model, and contour analysis is applied to each segmented part. The features from the RGB and depth streams are fused, and a Transformer-based architecture is used to capture temporal dynamics and assess exercise quality. The workflow is shown on Figure 1.

### 3.1 RGB-KPD

The methodology for extracting human silhouettes from video frames involves three core stages: preprocessing, semantic segmentation, and silhouette extraction. These operations are applied on a per-frame basis, assuming that frames have already been extracted from the input videos.

#### 3.1.1 Preprocessing

Each input frame, denoted as $I_{frame}\left(x,y\right)$ , where $\left(x,y\right)$ are pixel coordinates, is initially converted from BGR to RGB color space. It is then resized to match the input dimensions expected by the semantic segmentation model, represented as $\left(W,H\right)$ . The resized image is normalized using the mean and standard deviation statistics of the model’s training dataset. Mathematically, this transformation can be written as in Equation 1.

$$I_{preprocessed}\left(x,y\right)=\frac{I_{RGB}\left(x^{\prime },y^{\prime }\right)-\mu }{\sigma }$$ (1)

where I represent the input frame. (x, y) are the pixel coordinates in the resized image of dimensions H × W, $\left(x^{\prime },y^{\prime }\right)$ are the corresponding pixel coordinates in the original BGR image after resizing to H × W, $I_{RGB}\left(x^{\prime },y^{\prime }\right)$ denotes the RGB color value at pixel (x′,y′) after the BGR to RGB conversion and resizing, μ represents the mean vector (or scalar if it's a per-channel mean) calculated from the model’s training dataset and σ represents the standard deviation vector (or scalar if it's a per-channel standard deviation) calculated from the model’s training dataset. This normalized image is then converted into a tensor as in Equation 2.

$$I_{tensor}=Tensorize\left(I_{preprocessed}\right)$$ (2)

#### 3.1.2 Semantic segmentation

The tensor $I_{tensor}$ is passed through a DeepLabV3 (Hamamoto et al., 2024) segmentation model with a ResNet-101 (Panigrahi et al., 2024) backbone to produce a pixel-wise segmentation map. The model outputs a probability distribution for each pixel given in Equation 3.

$$S_{out}\left(x,y\right)=DeepLabV3\left(\;I_{tensor}\right)\;\left(x,y\right)$$ (3)

To assign a class label to each pixel, the class with the highest probability is selected using the argmax operation given by Equation 4.

$$S_{seg}\left(x,y\right)={\arg\max}_{n\epsilon \left\{1,\ldots ,N\right\}}S_{out}\left(x,y\right)\left[n\right]$$ (4)

where arg max selects the index n of the highest probability in the vector $S_{out}\left(x,y\right)$ corresponding to the class label assigned to that pixel. Following this, a binary mask $M_{raw}\left(x,y\right)$ is generated by analyzing all regions in $S_{seg}$ . Among all the segmented regions, only the largest connected component is retained, ensuring that the most prominent human figure in the frame is selected using Equation 5.

$$M_{raw}\left(x,y\right)=\left\{\begin{array}{l} 255,if\;\left(x,y\right)\in LargestComponent\left(S_{seg}\right)\\ 0,otherwise \end{array}\right.$$ (5)

#### 3.1.3 Silhouette extraction

To refine the extracted mask, morphological operations are applied to $M_{raw}\left(x,y\right)$ . Specifically, opening and closing operations are performed using a kernel K of size 5×5 to remove small artifacts and fill small holes given by Equation 6.

$$M_{cleaned}=\left(M_{raw}\circ K\right)\cdot K=\left[\left(M_{raw}⊖K\right)⊕K\right]$$ (6)

where $K=\left\{\left[\left(M_{raw}⊖K\right)⊕K\right]⊕K\right\}⊖K$ . The cleaned mask is then resized back to the original frame dimensions $\left(W,H\right)$ for accurate alignment using Equation 7.

$$M_{aligned}\left(x,y\right)=M_{cleaned}\left(\left\lfloor \frac{x.W^{\prime }}{W}\rfloor ,\lfloor \frac{y.H^{\prime }}{H}\right\rfloor \right)$$ (7)

where ⌊⋅⌋ denotes the floor function (used in nearest-neighbor interpolation), x∈[0, W−1], y∈[0, H−1]. Finally, the silhouette frame is generated by applying this mask to the original input frame. Only pixels corresponding to the detected human are retained, while all other pixels are set to zero (black background). The final silhouette frame $I_{silhouette}\left(x,y\right)$ is computed as in Equation 8.

$$I_{silhouette}\left(x,y\right)=\left\{\begin{array}{l} I_{frame}\left(x,y\right),if\;M_{aligned}\left(x,y\right)=255\\ 0,otherwise \end{array}\right.$$ (8)

This approach ensures the robust isolation of the human figure from each frame, producing clean silhouettes suitable for downstream analysis.

#### 3.1.4 2D keypoints feature extraction

To extract meaningful structural keypoints from binary human silhouettes, we employed a suite of classical keypoint detection techniques rooted in image geometry and intensity discontinuity. Each method targets distinct properties of the silhouette and collectively offers a diverse spatial representation of the human form across varying poses.

##### 3.1.4.1 Contour-based keypoints

**FIGURE 2:**
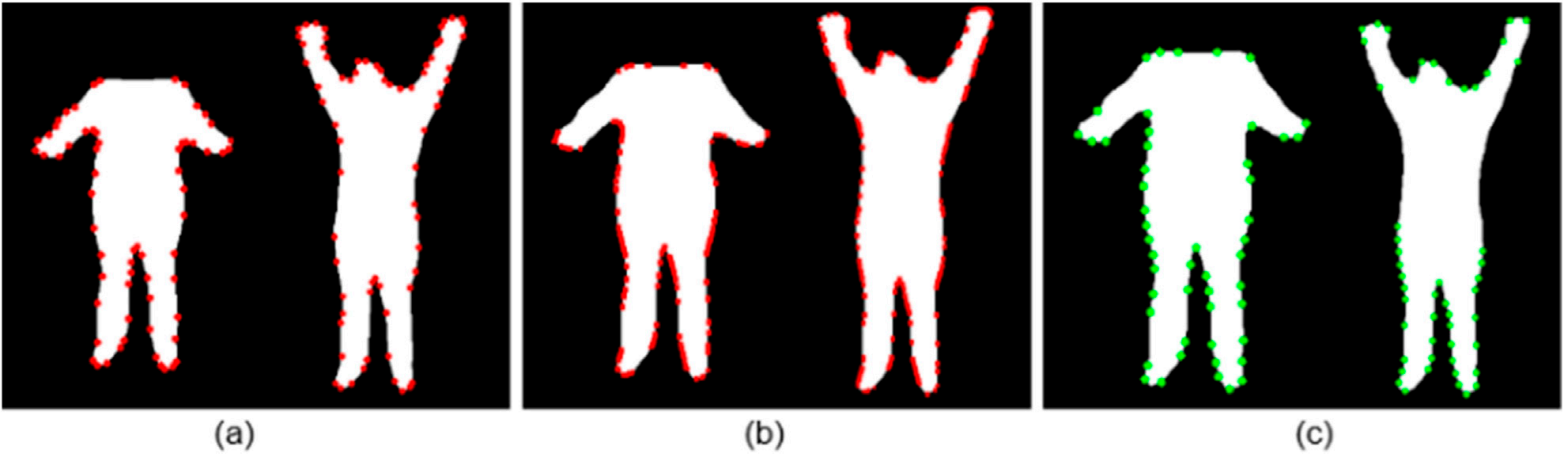
Keypoint detection on silhouette images using **(a)** Contour approximation, **(b)** Harris corner detection, and **(c)** Shi-Tomasi method.

Contour approximation detects the outer boundary of a shape and simplifies it into a polygonal representation. As illustrated in Figure 2a, this method localizes keypoints along the silhouette’s perimeter, concentrating on high-curvature regions such as elbows, knees, and shoulder angles. By adjusting the approximation tolerance, the method effectively balances geometric precision and sparsity, resulting in a reduced set of anatomically relevant points. The polygonal simplification is governed by the Douglas–Peucker algorithm (Douglas and Peucker, 1973), which recursively removes points where the perpendicular distance d⊥ to the baseline segment is below a specified threshold ε using Equation 9.

$$d⊥=\frac{\left|\left(x_{2}-x_{1}\right)\left(y_{1}-y_{0}\right)-\left(x_{1}-x_{0}\right)\left(y_{2}-y_{1}\right)\right|}{\sqrt{{\left(x_{2}-x_{1}\right)}^{2}+{\left(y_{2}-y_{1}\right)}^{2}}}$$ (9)

##### 3.1.4.2 Harris corner detection

Harris corner detection identifies regions with strong local intensity variations, mathematically characterized by eigenvalue analysis of the gradient covariance matrix (Harris and Stephens, 1988). In silhouette imagery, these variations are caused by shape discontinuities. As shown in Figure 2b, Harris keypoints are densely distributed around joint regions, including wrists, ankles, and neck transitions, where the silhouette contour exhibits abrupt directional changes. The corner response function is defined as in Equation 10.

$$R=\det\left(M\right)-k.{\left(trace\left(M\right)\right)}^{2}$$ (10)

where M is the second-moment matrix given in Equation 11.

$$M=\left[\begin{array}{cc} I_{x}^{2} & I_{X}I_{y}\\ I_{X}I_{y} & I_{y}^{2} \end{array}\right]$$ (11)

and $I_{X},I_{y}$ are image gradients along the x and y directions, respectively.

##### 3.1.4.3 Shi-Tomasi corner detection

Shi-Tomasi enhances the Harris approach by retaining only those points with the highest structural stability, as defined by the minimum eigenvalue of the autocorrelation matrix (Shi and Tomasi, 1994). As visualized in Figure 2c, this results in spatially clean and anatomically consistent points located primarily at prominent body joints.

##### 3.1.4.4 ORB-based detection

**FIGURE 3:**
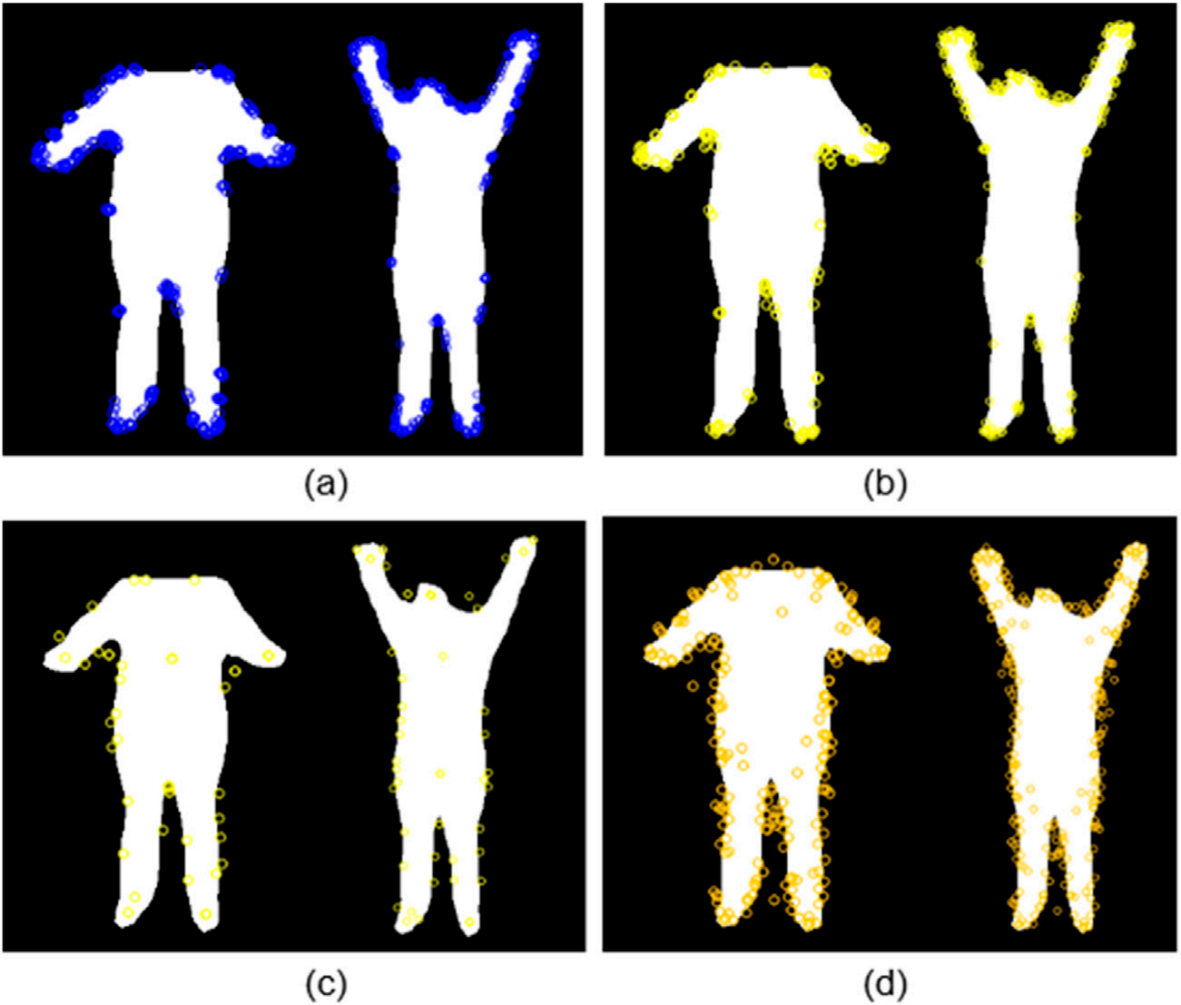
Keypoint detection results using **(a)** ORB, **(b)** BRISK, **(c)** SIFT, and **(d)** AKAZE on binary human silhouettes.

Oriented FAST and Rotated BRIEF (ORB) utilizes intensity difference testing over a circular neighborhood to identify stable keypoints (Rublee et al., 2011). As seen in Figure 3a, the detected points consistently emerge at limb extremities, head contours, and joint areas. ORB is particularly effective at capturing repeated spatial patterns across multiple poses, making it well-suited for silhouette-based action analysis.

##### 3.1.4.5 BRISK-based detection

Binary Robust Invariant Scalable Keypoints (BRISK) identifies local extrema by comparing intensity patterns across concentric circular layers (Leutenegger et al., 2011). Figure 3b demonstrates that BRISK effectively highlights joint-like structures and pose-specific inflection points such as raised hands, bent arms, and inclined postures. The circular sampling design contributes to its ability to adapt to shape deformation and body articulation.

##### 3.1.4.6 SIFT-based detection

SIFT (Scale-Invariant Feature Transform) identifies keypoints by locating extrema in scale-normalized Difference-of-Gaussian space (Lowe, 2004). Despite the lack of texture in silhouette images, the method succeeds in capturing stable points at scale-consistent curvature zones. As seen in Figure 3c, SIFT keypoints predominantly lie along the outer edges, providing a compact yet descriptive summary of the silhouette geometry. The scale-space extrema are located by solving using Equation 12.

$$\frac{\partial D\left(x,y,\sigma \right)}{\partial \sigma }=0$$ (12)

where $D\left(x,y,\sigma \right)=L\left(x,y,k\sigma \right)-L\left(x,y,\sigma \right)$ and L represents the Gaussian-blurred image at scale σ .

##### 3.1.4.7 AKAZE-based detection

Accelerated KAZE (AKAZE) operates in nonlinear scale space and extracts robust keypoints even under low contrast (Alcantarilla et al., 2011). Its performance on silhouette data is illustrated in Figure 3d, where keypoints are clustered around the torso and limbs. The method adapts well to body articulation and provides enhanced sensitivity to localized structural transitions.

##### 3.1.4.8 Skeleton-based landmark detection

**FIGURE 4:**
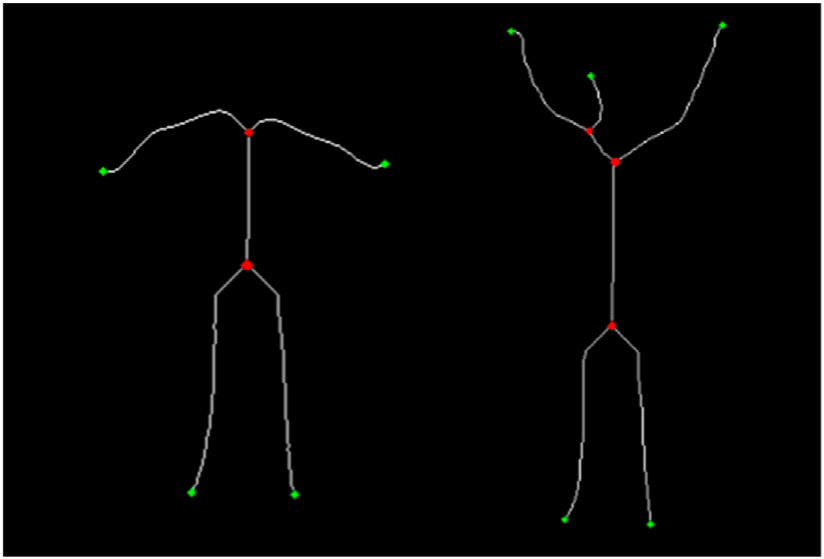
Skeleton-based keypoints showing endpoints (green) and branch points (red) on human silhouettes.

To extract topological keypoints, we applied skeletonization to reduce each silhouette to its medial axis (Zhang and Suen, 1984). Endpoints and branch points were identified by analyzing the neighborhood connectivity of skeletal pixels. As shown in Figure 4, this method reliably identifies semantically meaningful regions such as fingertips, feet, and limb-torso junctions, offering a structural representation aligned with human pose semantics.

#### 3.1.5 Body part labelling

To derive a semantically rich, region-specific understanding of the human body, we employed a body part labeling (BPL) approach using the Single-Human-Parsing-LIP (Huang and Yang, 2024) model proposed by Huang et al. This model, based on a deep convolutional encoder-decoder framework,\ performs dense pixel-wise classification across twenty predefined body parts including limbs, torso, and accessories. Due to its relatively lightweight architecture and efficient inference capability, it serves as a computationally economical solution well-suited for large-scale or resource-constrained deployments.

**FIGURE 5:**
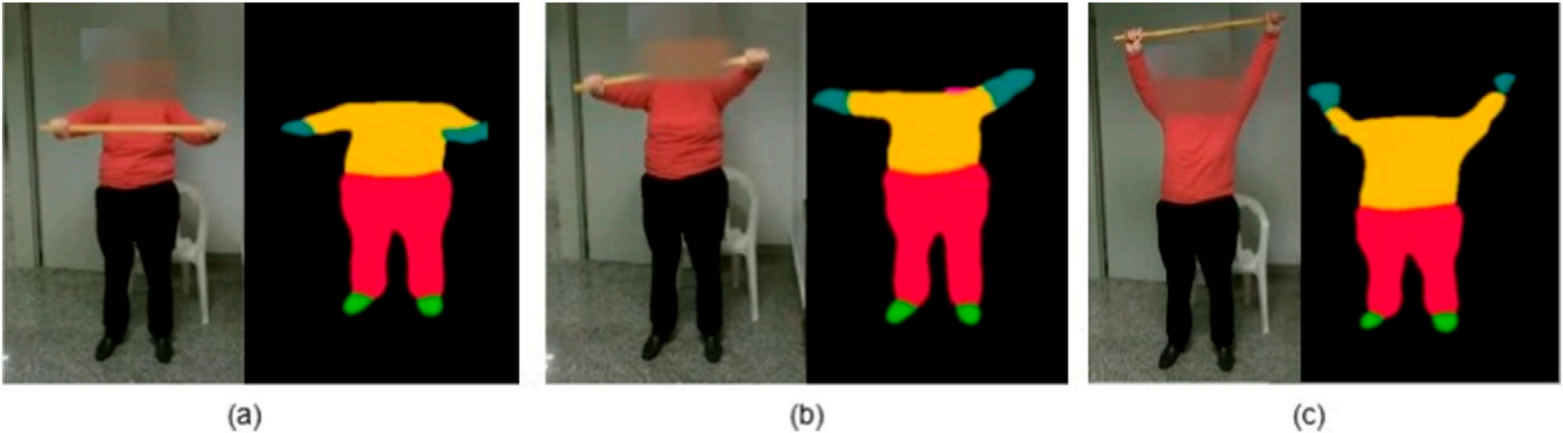
Semantic segmentation results using the LIP model for different upper body poses: **(a)** Arms extended at chest level, **(b)** Arms extended at head level, **(c)** Arms raised above the head.

**FIGURE 6:**
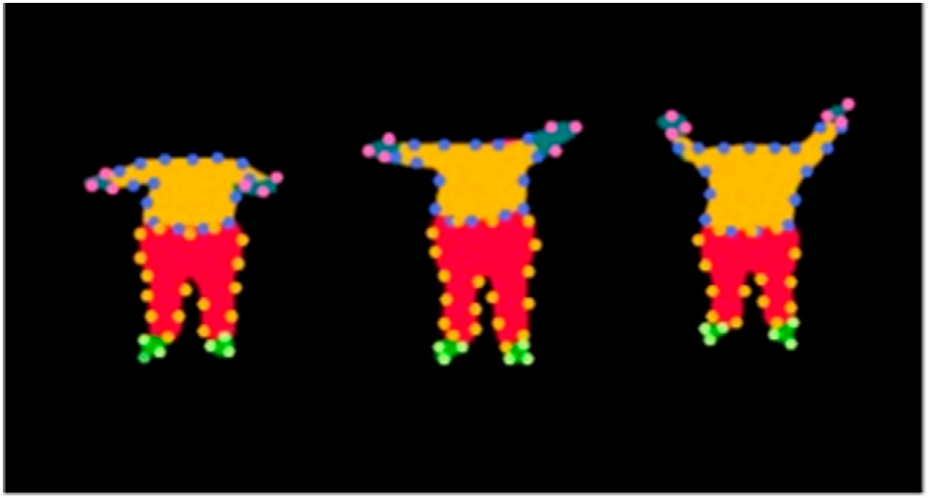
Part-wise contour point visualization across various frames.

The model was applied on preprocessed silhouette frames to generate multi-class segmentation masks where each pixel is mapped to a corresponding anatomical region. Specifically, the model produces a per-pixel probability distribution ${\hat{Y}}_{i,j}=\left[{{\hat{y}}_{i,j}}^{\left(1\right)},{{\hat{y}}_{i,j}}^{\left(2\right)},\ldots ,{{\hat{y}}_{i,j}}^{\left(N\right)}\;\right]$ , and the final label map $L\in Z^{H\times W}$ is obtained by Equation 13.

$$L_{i,j}=\underset{c\in \left\{1,\ldots ,N\right\}}{\mathrm{argmax}}{{\hat{y}}_{i,j}}^{\left(C\right)}$$ (13)

where ${{\hat{y}}_{i,j}}^{\left(C\right)}$ denotes the predicted probability of class ccc at pixel $\left(i,j\right)$ . As shown in Figure 5, each segmented region is color-coded for visual clarity, facilitating subsequent part-wise analysis. We developed a color-guided contour extraction method using the semantic label map from the parsing model. Each body part was isolated with its unique color, followed by binary masking and intensity thresholding for boundary extraction. Contours were traced with a point-based algorithm and visualized with color-coded hexagonal markers to represent anatomical regions, as shown in Figure 6. This approach effectively captures geometric structures aligned with human anatomy.

### 3.2 D-mesh

In D-Mesh phase, the methodology for processing depth images involves several stages: preprocessing and Dynamic KeyPoint Network (DKP-Net) for keypoint extraction. Preprocessing enhances the image by removing noise, the floor, and improving contrast. DKP-Net extracts 3D keypoints, capturing x, y coordinates, and z-depth. The 3D body joint positions extracted are passed to the Skinned Multi-Person Linear (SMPL) model to generate detailed 3D body mesh vertices, along with pose and shape parameters. DKPNet uses tailored pipelines (DKP-Net-24-L and DKP-Net-24-R) for different arm positions.

#### 3.2.1 Preprocessing

This study employs the RANSAC algorithm to effectively remove the floor from the depth image by fitting a plane model to the detected floor points. The process starts by identifying floor pixels-based on their depth values and using a binary mask to ensure that only foreground pixels are considered. The depth values are then analyzed to generate a set of 3D points representing the floor, as defined in Equation 14.

$$P\_floor=\left\{\left(x,y\right)\left|z\right.>0\;and\;binary\_m\left[y,x\right]=255\right\}$$ (14)

where z represents the depth value, and the binary mask is used to differentiate foreground pixels from background pixels. The RANSAC algorithm is then applied to estimate a planar model that best fits the floor points, as described by Equation 15.

z=a.x+b.y+c (15)

After computing the floor model, points with residuals smaller than a predefined threshold ϵ are identified as floor pixels and removed, as illustrated in Equation 16.

$$Depth_{corrected\left[y,x\right]}=\left\{\begin{array}{l} 0,\left|z-a.x+b.y+c\right|<\varepsilon \\ z,otherwise \end{array}\right.$$ (16)

**FIGURE 7:**
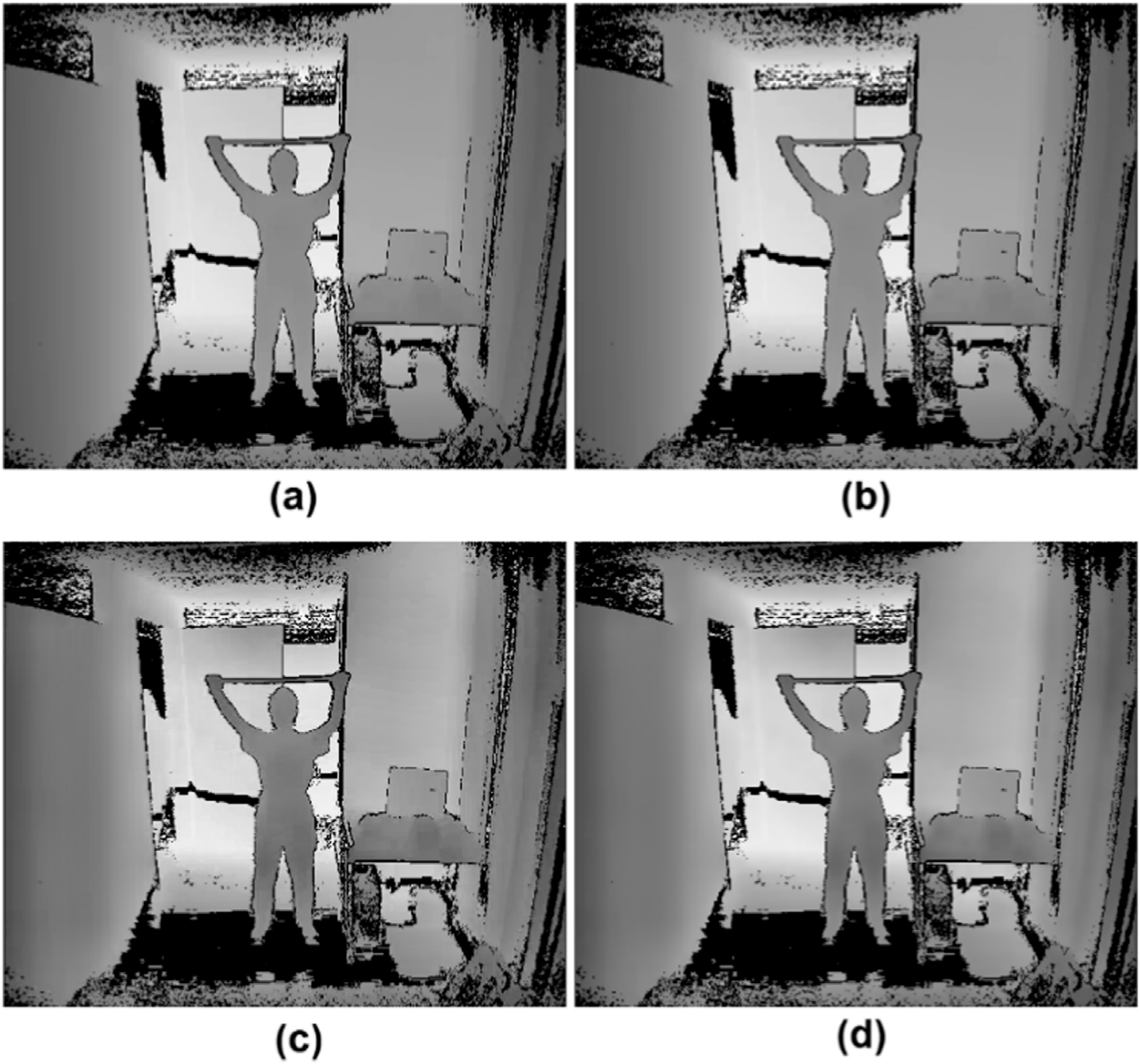
Preprocessing steps: **(a)** Floor-removed image, **(b)** Normalization, **(c)** CLAHE, and **(d)** Bilateral Filtering.

This process effectively removes the floor while maintaining the structural integrity of the other depth values in the image, as depicted in Figure 7a. The depth image, with the floor removed, $I_{d}$ , is then normalized to enhance contrast using Min-Max normalization using Equation 17, as described in Figure 7b.

$$I_{norm}\left(x,y\right)=\frac{I_{d}\left(x,y\right)-\min\left(I_{d}\right)}{\max\left(I_{d}\right)-\min\left(I_{d}\right)}\times 255$$ (17)

To further enhance contrast, we apply CLAHE (Contrast Limited Adaptive Histogram Equalization), which improves local contrast while avoiding excessive noise amplification as shown in Figure 7c. The transformation is described by Equation 18, where $P_{clip}\left(i\right)$ represents the clipped cumulative distribution function used in CLAHE.

$$I_{clahe}\left(x,y\right)=\sum_{i=0}^{I_{norm}\left(x,y\right)}P_{clip}\left(i\right)$$ (18)

To make the grayscale depth image compatible with color-based processing techniques, we duplicate the single channel across three channels, as shown in Equation 19.

$$I_{3\text{ch}}={\left(I_{clahe}\left(x,y\right),I_{clahe}\left(x,y\right),I_{clahe}\left(x,y\right)\right)}^{T}$$ (19)

To preserve edges while minimizing noise, we apply bilateral filtering using Equation 20, as described in as shown in Figure 7d.

$$I_{filtered}\left(x,y\right)=\frac{\sum_{xi,yi}I_{3ch}\left(xi,yi\right)G_{s}\left(d\right)G_{r}\left(r\right)}{\sum_{xi,yi}G_{s}\left(d\right)G_{r}\left(r\right)}$$ (20)

where d represents the spatial distance, rrr represents the intensity difference, and $G_{s}$ , $G_{r}$ are Gaussian functions. These preprocessing steps are illustrated in Figure 7.

#### 3.2.2 Deep learning-based human silhouette isolation

To segment objects from depth images, we apply the DeepLabV3+ segmentation model with a ResNet-101 backbone, as illustrated in Figure 4. This model uses Atrous Spatial Pyramid Pooling (ASPP) to capture multi-scale contextual information. The preprocessed image is resized to 256 × 256 pixels and then transformed into a tensor, as defined in Equation 8, where T represents the tensor transformation and R denotes the resizing operation given by Equation 21.

$$T\left(x,y\right)=T\left(R\left(I_{filtered}\times 256\times 256\right)\right)$$ (21)

The transformed image tensor is input into the DeepLabV3+ model, which produces a pixel-wise segmentation map, as shown in Equation 22.

$$P\left(x,y\right)=\arg\max_{C}M_{out}\left(x,y,c\right)$$ (22)

Morphological operations refine the segmentation mask through resizing, closing, and dilation, ensuring precise segmentation for applications like activity recognition and medical imaging.

#### 3.2.3 Dynamic Keypoint Network – 24 points (DKP-Net-24)

DKP-Net-24 (Dynamic Keypoint Network – 24 Points) is a robust framework for extracting keypoints from depth-based silhouettes. Unlike static methods, it dynamically adjusts to variations in pose, body alignment, and arm positions, making it ideal for motion tracking and rehabilitation assessment. The system uses two pipelines to extract 3D keypoints (x, y, z) for detailed human motion representation. DKP-Net-24-L handles lowered arms using contour-based analysis, while DKP-Net-24-R is optimized for raised arms, ensuring reliable keypoint detection. The extraction procedure for lowered arms is outlined in Table 1.

**TABLE 1:** (DKP-Net-24-L) keypoint detection for lowered arms.

1. procedure MAIN(human_silhouette)
2. Initialize an empty list for results
results ← []
3. Set the input silhouette
silhouette ← human_silhouette
4. Determine the initial position of the head
head_x, head_y ← width/2, top_pixel_y
5. Set the neck position just below the head
neck_x, neck_y ← width/2, head_y + h/8
6. Find the x-coordinate of the right shoulder at the neck level
shoulder_right_x ← max{x | silhouette(neck_y, x) > 0} - 5
7. Find the x-coordinate of the left shoulder at the neck level
shoulder_left_x ← min{x | silhouette(neck_y, x) > 0} + 5
8. Calculate the center x-coordinate of the neck between the shoulders
neck_x ← avg(shoulder_right_x, shoulder_left_x)
9. Adjust collarbone positions based on shoulder coordinates
collarbone_left_x, collarbone_right_x ← (shoulder_left_x + 15, shoulder_right_x - 15)
10. Define the starting and ending positions of the hips
hip_start, hip_end ← 3h/5, 2h/3
11. Find the left and right hip positions
hip_left_x, hip_right_x ← min/max{x | silhouette(y, x) > 0, y ∈ [hip_start, hip_end]}
12. Calculate the pelvis position
pelvis_x, pelvis_y ← avg(hip_left_x, hip_right_x), avg(hip_start, hip_end)
13. Determine the center of the spine
spine_x, spine_y ← avg(neck_x, pelvis_x), avg(neck_y, pelvis_y)
14. Define the upper spine position
spine_upper_x, spine_upper_y ← avg(neck_x, spine_x), avg(neck_y, spine_y)
15. Define the lower spine position
spine_lower_x, spine_lower_y ← avg(pelvis_x, spine_x), avg(pelvis_y, spine_y)
16. Find the elbow positions based on the neck and spine range
elbow_left_x, elbow_right_x ← min/max{x | silhouette(y, x) > 0, y ∈ [neck_y, spine_y]}
17. Find the wrist positions based on the spine and pelvis range
wrist_left_x, wrist_right_x ← min/max{x | silhouette(y, x) > 0, y ∈ [spine_y, pelvis_y]}
18. Locate the ankle positions at the bottom of the silhouette
ankle_left_x, ankle_right_x ← min/max{x | silhouette(bottom_pixel_y, x) > 0}
19. Adjust heel positions slightly lower than the ankles
heel_left_y, heel_right_y ← ankle_left_y - h/40, ankle_right_y - h/40
20. Display the detected keypoints
results ← DISPLAY_RESULTS(all keypoints)
return results
21. end procedure

**FIGURE 8:**
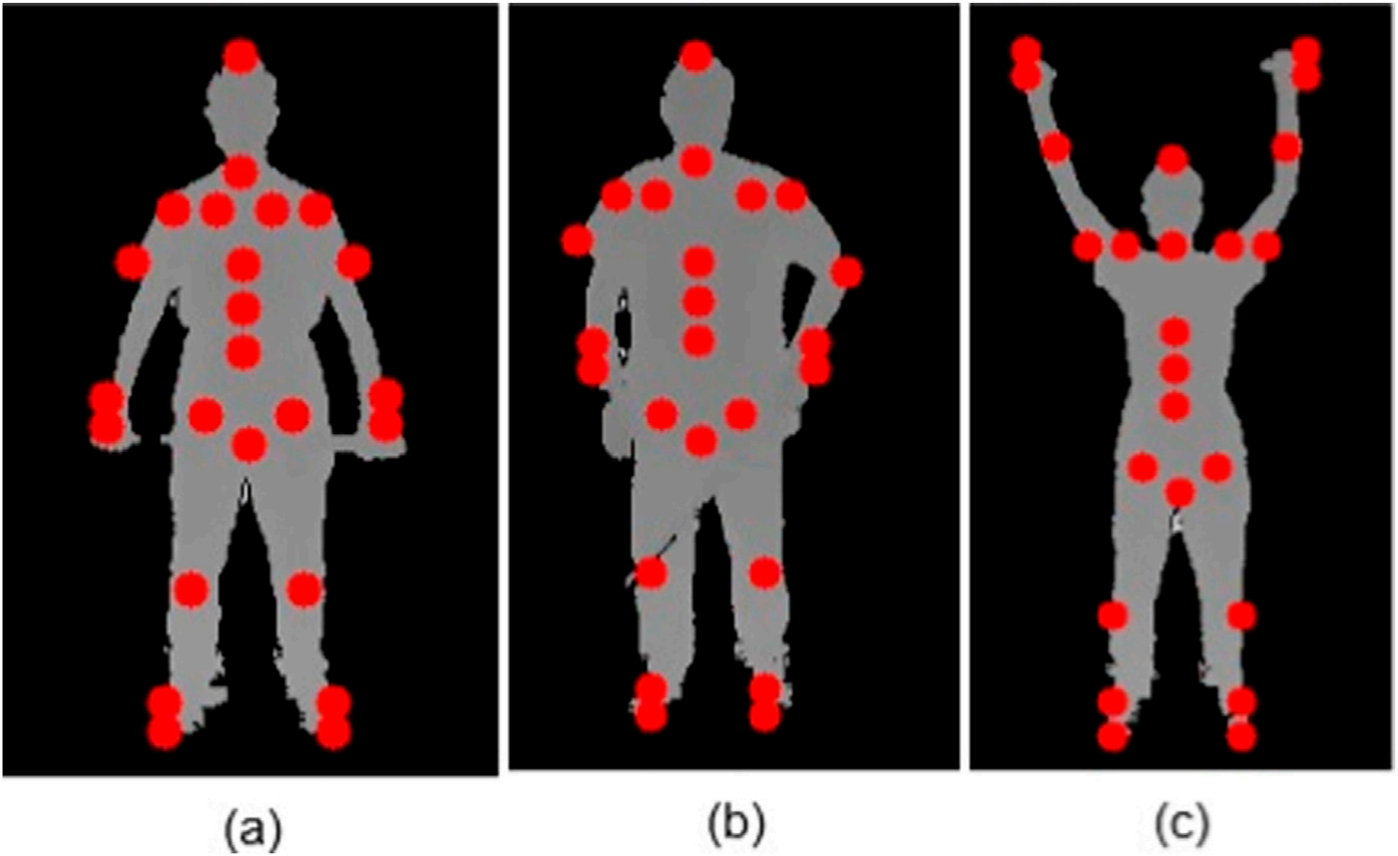
Detected key body points for different postures: **(a)** Neutral stance, **(b)** One-arm relaxed, and **(c)** Arms raised. When the arms are raised above shoulder level, the algorithm described in Table 2 adapts the keypoint localization process to ensure accurate tracking of the shoulders, wrists, and hand.

When the arms are raised above shoulder level, the algorithm described in Table 2 adapts the keypoint localization process to ensure accurate tracking of the shoulders, wrists, and hand. Detected key body points for different postures are shown in Figure 8.

**TABLE 2:** (DKP-Net-24-R) keypoint detection for raised arms.

1. procedure MAIN(human_silhouette)
2. Initialize an empty list for results
results ← []
3. Set the silhouette as input human silhouette
silhouette ← human_silhouette
4. Define hip start and end positions based on height
hip_start, hip_end ← (3h/5, 2h/3)
5. Calculate the x-coordinates of the left and right hips
hip_left_x, hip_right_x ← min/max{x | silhouette(y, x) > 0, y ∈ [hip_start, hip_end]}
6. Calculate the pelvis center coordinates
pelvis_x, pelvis_y ← avg(hip_left_x, hip_right_x), avg(hip_start, hip_end)
7. Set body center positions for the left and right sides
body_center_left, body_center_right ← hip_left_x, hip_right_x
8. Find the head position based on the first non-zero pixel above the hips
head_x, head_y ← first nonzero pixel in [0, hip_start]
9. Calculate the neck position by offsetting the head’s position
neck_x, neck_y ← (head_x, head_y + h/12)
10. Identify the x-coordinates of the left and right shoulders at the neck level
shoulder_left_x, shoulder_right_x ← min/max{x | silhouette(neck_y, x) > 0} ± 5
11. Adjust collarbone positions from the shoulders
collarbone_left_x, collarbone_right_x ← (shoulder_left_x + 15, shoulder_right_x - 15)
12. Determine the left and right hand x-coordinates based on silhouette across columns
left_hand_x, right_hand_x ← min/max{x | silhouette[:, x].any()}
13. Set wrist coordinates based on hand positions with a slight vertical offset
left_wrist_x, right_wrist_x ← (left_hand_x, right_hand_x), (left_hand_y + 10, right_hand_y + 10)
14. Locate the elbow positions between the neck and spine regions
elbow_left_x, elbow_right_x ← min/max{x | silhouette(y, x) > 0, y ∈ [neck_y, spine_y]}
15. Calculate the center of the spine by averaging pelvis and neck coordinates
spine_x, spine_y ← avg(neck_x, pelvis_x), avg(neck_y, pelvis_y)
16. Define the upper spine coordinates between neck and spine centers
spine_upper_x, spine_upper_y ← avg(neck_x, spine_x), avg(neck_y, spine_y)
17. Locate the knee positions around 3/4 of the height from the top
knee_y ← 3h/4, knee_left_x, knee_right_x ← min/max{x | silhouette(knee_y, x) > 0} ± 7
18. Find the ankle positions at the bottom of the silhouette
ankle_left_x, ankle_right_x ← min/max{x | silhouette(bottom_pixel_y, x) > 0}
19. Adjust heel positions slightly lower than the ankles
heel_left_y, heel_right_y ← ankle_left_y - h/30, ankle_right_y - h/30
20. Display all detected keypoints
results ← DISPLAY_RESULTS(all keypoints)
21. Return the final results
return results
22. end procedure

#### 3.2.4 3D mesh reconstruction

In this work, we developed a pipeline for 3D human mesh reconstruction and SMPL model fitting (Loper et al., 2015) using multiple motion capture datasets. To ensure compatibility across different skeletal formats, we applied a joint mapping strategy that converts our DKP-Net-24 joint extraction into the SMPL structure. These joints are then used to estimate 3D poses and reconstruct body geometry. The SMPL model provides a learned, parametric mesh representation with 6,890 vertices and 13,776 faces, enabling realistic and efficient modeling for animation and analysis.

This model defines the human body through two parameter sets: pose parameters $\theta \in R^{72}$ , which encode 3D axis-angle rotations across 24 joints, and shape parameters $\beta \in R^{10}$ which describe identity-specific body shape variations based on a low-dimensional shape space derived from body scan datasets. The SMPL mesh is computed through a blend function that incorporates these parameters using Equation 23.

$$M\left(\theta ,\beta \right)=W\left(T\left(\theta ,\beta \right),J\left(\beta \right),\theta ,\omega \right)$$ (23)

where $T\left(\theta ,\beta \right)$ denotes the template mesh deformed by shape and pose, $J\left(\beta \right)$ represents joint locations derived from the shape-dependent skeleton, $W\left(.\right)$ is the linear blend skinning (LBS) function, applying the rotations defined by θ using precomputed weights ω. The model outputs three key components: the vertex positions $V\in R^{6890\times 3}$ that define the surface geometry, the joint positions $J\in R^{24\times 3}$ used for pose tracking, and the face connectivity $F\in R^{13776\times 3}$ which defines the mesh structure. To align with the SMPL model, the 24 joints are structured as (frames, 24, 3) tensors. A depth inversion corrects orientation, and one-to-one mapping ensures anatomical alignment. SMPL fitting minimizes joint loss by reducing the Euclidean distance between predicted and extracted joints using Equation 24.

$$L_{joint}=\sum_{i=1}^{24}\omega_{i}{\left\|J_{SMPL}-J_{computed}\right\|}_{2}^{2}$$ (24)

where $\omega_{i}$ are per-joint weights that control the importance of each joint in the loss calculation. To ensure physiologically plausible poses, a probabilistic prior from a Gaussian Mixture Model (GMM) trained on real motion data is used. It penalizes poses that deviate from natural human movement patterns using Equation 25.

$$L_{pose}=-\log p\left(\theta \right)$$ (25)

where p(θ) is the GMM likelihood, a regularization term penalizes extreme shape values to ensure realistic body proportions using Equation 26.

$$L_{shape}={\left\|\beta \right\|}_{2}^{2}$$ (26)

A smoothness constraint is added to ensure continuity between frames, reducing jitter by penalizing large joint position changes between consecutive frames using Equation 27.

$$L_{smooth}=\sum_{t=1}^{T-1}{\left\|J_{SMPL}-J_{computed}\right\|}_{2}^{2}$$ (27)

where T is the number of frames, Laplacian regularization ensures smooth mesh surfaces by keeping vertices near their neighbors using Equation 28.

$$L_{mesh}=\sum_{i}{\left\|V_{i}-\frac{1}{\left|N_{i}\right|}\sum_{j\in N_{i}}V_{j}\right\|}_{2}^{2}$$ (28)

In this expression, $V_{i}$ is the position of the *i*th vertex and $N_{i}$ denotes its one-ring neighborhood. The overall objective function combines these components, with each term weighted by a corresponding coefficient $\lambda_{i}$ to control its influence using Equation 29.

$$L_{total}=\lambda_{1}L_{joint}+\lambda_{2}L_{pose}+\lambda_{3}L_{shape}+\lambda_{4}L_{smooth}+\lambda_{5}L_{mesh}$$ (29)

**FIGURE 9:**
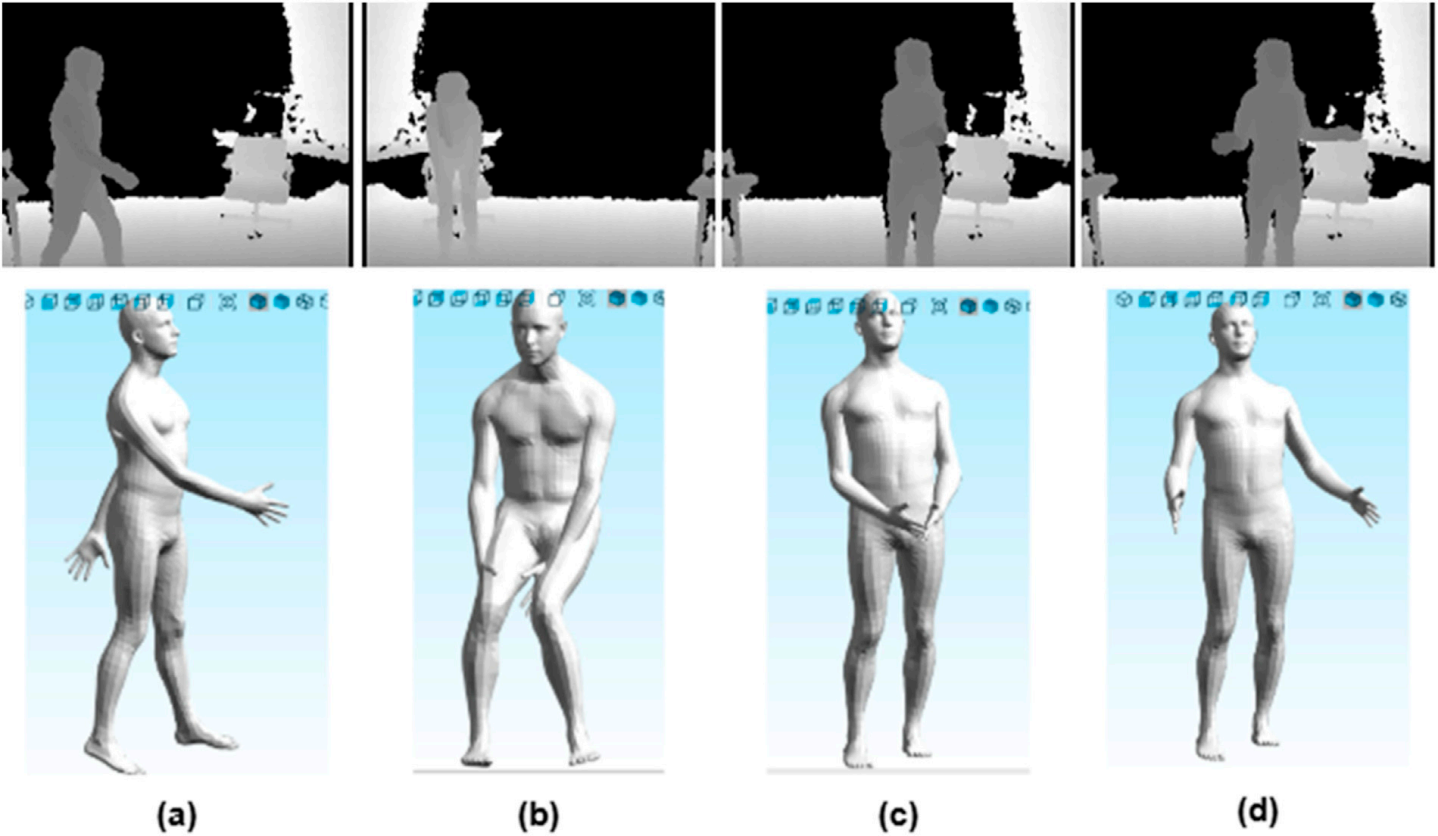
3D reconstruction of a subject from the UTKinect-Action3D dataset: **(a)** Random walk toward the chair **(b)** Leaning forward to pick up an object with both hands closing **(c)** Closing of arms **(d)** Opening the arms after the clap.

**FIGURE 10:**
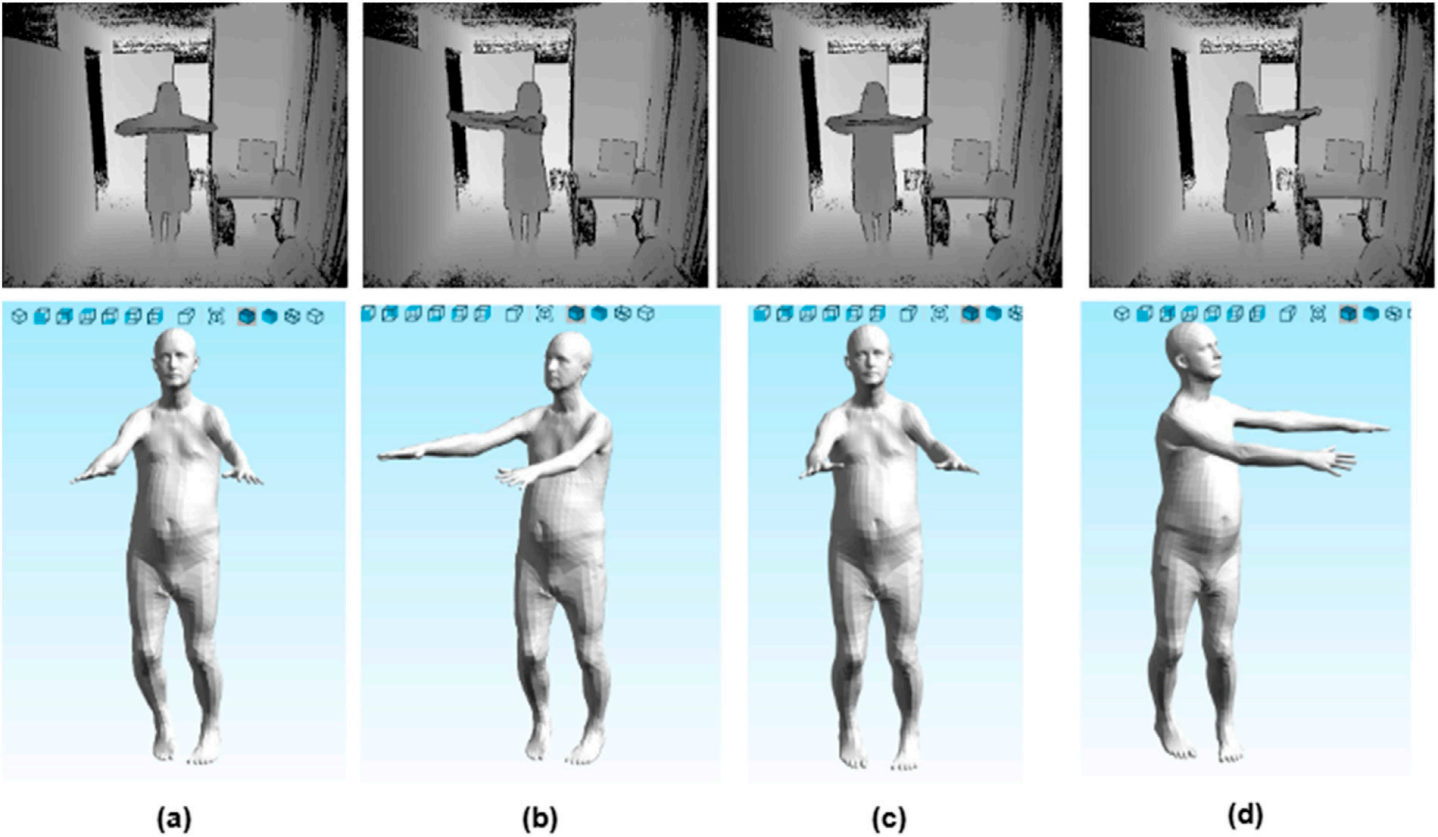
3D reconstruction of a subject from the KIMORE dataset: **(a)** Holding a bar with both hands at face level **(b)** Moving the bar to the right side while keeping it in both hands **(c)** Bringing the bar back to the front of the face **(d)** Moving the bar to the left side while keeping it in both hands.

The loss is minimized with Adam optimization, refining θ and β for accurate 3D meshes. Figures 9, 10 show depth frames and 3D meshes for body poses from KIMORE and UTKinect-Action3D datasets.

### 3.3 Feature fusion

A feature fusion stage combines RGB and depth modalities. Specifically, let $k_{i}\epsilon R^{P}$ represent keypoint features from the *i*th RGB frame, capturing visual cues through methods like contour approximation, corner detection (e.g., Harris, Shi-Tomasi), and feature descriptors (e.g., ORB, SIFT). Furthermore, let $b_{i}\epsilon R^{Q}$ denote body part label features from depth data, encoding segmentation and body region identification. In addition, $m_{i}\epsilon R^{S}$ represents 3D mesh parameters, including pose and shape, derived from depth data using models like SMPL. The fused feature vector $f_{i}$ is computed by concatenating these vectors using Equation 30.

$$f_{i}=k_{i}⊕b_{i}⊕m_{i}$$ (30)

where ⊕ represents the concatenation operation, resulting in $f_{i}\epsilon R^{P+Q+S}$ . Fusion of RGB and depth enhances robustness to occlusion, clothing, and viewpoint changes. The fused feature vectors $f_{i}$ for a sequence of *M* frames are then assembled into a feature matrix $F=\left[f_{1},f_{2},\ldots ,f_{M}\right]\epsilon R^{M\times (P+Q+S)}$ , which serves as input to the subsequent temporal modeling stage for exercise recognition.

### 3.4 Transformer-based human action recognition

Given a sequence of fused numerical features for human action recognition, each time step $x_{t}\epsilon R^{D}$ encapsulates a combination of modalities such as spatial skeleton data, inertial sensor signals, and appearance features, all merged into a unified vector. This results in an input sequence $X=\left\{x_{1},x_{2},\ldots ,X_{T}\right\}$ , where T denotes the number of temporal frames, and each $x_{t}$ carries rich multimodal contextual information. The fused input is projected into a common latent space using a learnable transformation given by Equation 31.

$$e_{t}=W_{in}x_{t}+b_{in},e_{t}\epsilon R^{d_{model}}$$ (31)

To capture temporal ordering, positional encodings $p_{t}\epsilon R^{d_{model}}$ are added, yielding the input embeddings to the Transformer using Equation 32.

$$z_{t}^{\left(0\right)}=e_{t}+p_{t}$$ (32)

The resulting sequence $Z^{\left(0\right)}=\left\{z_{1}^{\left(0\right)},z_{2}^{\left(0\right)},\ldots ,z_{T}^{\left(0\right)}\right\}$ is passed to a stack of Transformer encoder layers, which learn attention-based temporal representations from the fused features. Inside each encoder layer, the Multi-Head Self-Attention mechanism enables the model to weigh interactions between time steps. For each layer, query, key, and value matrices are computed using Equation 33.

$$Q=ZW^{Q},K=ZW^{K},V=ZW^{V}$$ (33)

The scaled dot-product attention computes dynamic temporal dependencies using Equation 34.

$$Attention\left(Q,K,V\right)=softmax\left(\frac{QK^{T}}{\sqrt{d_{k}}}\right)V$$ (34)

In the multi-head form, multiple such attention mechanisms run in parallel using Equation 35.

$$MHSA\left(Z\right)=Concat\left(head_{1},\ldots ,head_{h}\right)W^{O}$$ (35)

This output is passed through a position-wise feed-forward network using Equation 36.

$$FFN\left(x\right)=\max\left(0,xW_{1}+b_{1}\right)W_{2}+b_{2}$$ (36)

Residual connections and normalization are applied to preserve gradients and stabilize learning using Equation 37.

$$Z^{\prime }=LayerNorm\left(Z+MHSA\left(Z\right)\right),Z^{\left(l+1\right)}=LayerNorm\left(Z^{\prime }+FFN\left(Z^{\prime }\right)\right)$$ (37)

**FIGURE 11:**
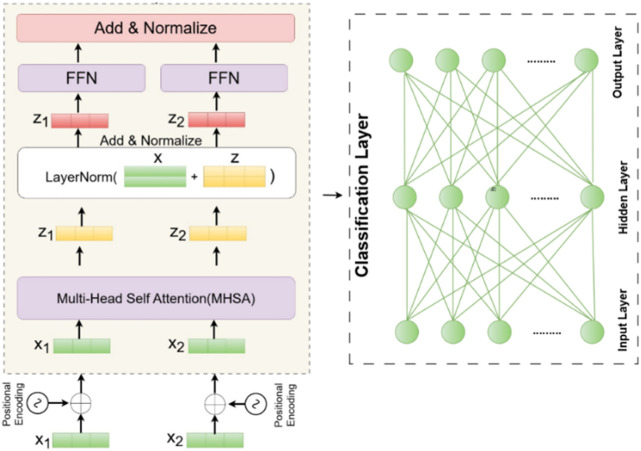
Illustration of the architecture of a Transformer encoder followed by a classification layer.

This pooled vector is then passed into a fully connected classification layer followed by a softmax to predict the action label using Equation 38.

$$y=softmax\left(W_{c}z_{agg}+b_{c}\right)$$ (38)

where, $y\epsilon R^{K}$ represents the probability distribution across human action classes. The model uses cross-entropy loss for optimization. The Transformer on fused features enhances accuracy by capturing spatial-temporal dependencies and multimodal complementarity. The workflow is in Figure 11, and the algorithm is in Table 3.

**TABLE 3:** 3D-poseformer: Multimodal-depth exercise recognition via 3D-mesh and Transformer.

3D-PoseFormer: Multimodal RGB-Depth Exercise Recognition via 3D Mesh and Transformer
Input: RGB_image, Depth_image
Output: exercise_label: Recognized exercise class
Algorithm
1. procedure MAIN(RGB_image, Depth_image)
2. rgb_features ← PROCESS_RGB(RGB_image)
3. depth_features ← PROCESS_DEPTH(Depth_image)
4. fused_features ← FUSE_FEATURES(rgb_features, depth_features)
5. predictions ← TRANSFORMER_CLASSIFIER(fused_features)
6. return predictions
7. end procedure
8. procedure PROCESS_RGB(image)
9. preprocessed ← PREPROCESS_RGB(image)
10. silhouette ← SEGMENT_HUMAN(preprocessed)
11. keypoints ← EXTRACT_2D_KEYPOINTS(silhouette)
12. part_labels ← BODY_PART_PARSING(silhouette)
13. contour_points ← EXTRACT_CONTOUR(part_labels)
14. return CONCAT(keypoints, contour_points)
15. end procedure
16. procedure PROCESS_DEPTH(image)
17. cleaned ← PREPROCESS_DEPTH(image)
18. silhouette ← SEGMENT_HUMAN_DEPTH(cleaned)
19. keypoints_3D ← EXTRACT_3D_KEYPOINTS(silhouette)
20. mesh ← RECONSTRUCT_3D_MESH(keypoints_3D)
21. smpl_params ← FIT_SMPL(mesh, keypoints_3D)
22. return smpl_params
23. end procedure
24. procedure FUSE_FEATURES(rgb_feats, depth_feats)
25. fused ← CONCAT(rgb_feats, depth_feats)
26. return fused
27. end procedure
28. procedure TRANSFORMER_CLASSIFIER(features)
29. embedded ← EMBED(features)
30. positional ← ADD_POSITIONAL_ENCODING(embedded)
31. for each layer in TRANSFORMER_ENCODER_STACK do
32. positional ← TRANSFORMER_ENCODER(positional)
33. end for
34. output ← CLASSIFY(positional)
35. return output
36. end procedure

## 4 Results and evaluation

### 4.1 Experimental setup

Complete implementation was conducted on a Google Colab virtual machine with an NVIDIA Tesla T4 GPU with 16 GB GDDR6 VRAM, 2,560 CUDA cores, and 320 Tensor cores, running Ubuntu 18.04.6 LTS. The environment used Python 3.10.13 with PyTorch 2.1.0+cu118 and cuDNN 8.9.1, enabling GPU-accelerated tensor computations and convolution operations. We used TorchVision 0.16.0 for image processing, NumPy 1.25.0 and Pandas 2.1.1 for data handling, and Matplotlib 3.8.0 and Seaborn 0.12.3 for visualization.

### 4.2 Datasets

We used the KIMORE dataset, which includes data from 78 subjects (44 healthy, 34 with low-back pain) performing five rehabilitation exercises. It provides RGB and depth videos, 25-joint skeleton positions, and clinical scores for each repetition, supporting intelligent remote rehabilitation monitoring. Additionally, we incorporated the mRI dataset, a multi-modal 3D pose estimation resource with over 5 million frames from 20 subjects, captured using RGB-D cameras, mmWave radar, and IMUs. This dataset aims to advance home-based health monitoring. Further to test the generalizability we thoughtfully selected UTKinect-Action3D action recognition dataset. The dataset records 10 subjects performing 10 daily-life actions, with synchronized RGB, depth, and skeletal data for generalizable action recognition in physical therapy.

The presented work is motivated by the rehabilitation of patients with lower back pain (LBP), and the KIMORE dataset directly reflects this scenario through rehabilitation-specific exercises performed by both healthy subjects and LBP patients. To complement this, we included the mRI and UTKinect-Action3D datasets to strengthen the generalization of the rehabilitation framework. The mRI dataset contains multi-modal recordings of repetitive and complex movements (e.g., bending, squatting, reaching), which closely resemble the functional motions targeted in LBP rehabilitation. Similarly, the UTKinect-Action3D dataset includes a wide variety of full-body actions that involve spinal mobility and trunk stability (e.g., bending, lifting, side movements), both of which are central components in evaluating rehabilitation progress for lower back disorders. By training and validating our model on these datasets, we ensure that the system is not overfitted to one rehabilitation dataset but can generalize to broader variations of human motion that are highly relevant to lower back rehabilitation tasks.

### 4.3 Confusion matrices

Table 4 shows the confusion matrix for correctness classification on the KIMORE dataset, achieving a 94.73% overall accuracy. Exercises E1 (0.92), E4 (0.97), and E5 (0.97) were classified highly accurately. Minor misclassifications occurred between E2 and E3, likely due to similar movement patterns.

**TABLE 4:** Confusion matrix for correctness classification for KIMORE dataset.

Class	E1	E2	E3	E4	E5
E1	0.92	0.03	0.03	0.01	0.02
E3	0.02	0.93	0.01	0.00	0.04
E3	0.02	0.06	0.88	0.02	0.02
E4	0.01	0.01	0.01	0.97	0.00
E5	0.02	0.00	0.01	0.01	0.97
Accuracy			**94.73%**		

Table 5 presents the confusion matrix for the mRI dataset, with a 91% overall accuracy across 12 exercise classes. Exercises such as E2, E3, E5, E6, E7, E9, E10, E11, and E12 showed excellent recognition (≥0.86). Some confusion occurred, notably for E1 and E4, due to overlapping execution characteristics. Table 6 shows results for the UTKinect-Action3D dataset, achieving 94.2% overall accuracy. Actions like Clap Hands (0.98), Wave Hands (0.97), Pick Up (0.97), and Throw (0.91) were classified with high precision. Minor confusion appeared between motion-similar actions like Stand Up and Walk and Carry and Pull. Overall, the model demonstrated strong classification performance across all three datasets, with most errors arising from visually or kinematically similar actions.

**TABLE 5:** Confusion matrix for correctness classification for mRI dataset.

Class	E1	E2	E3	E4	E5	E6	E7	E8	E9	E10	E11	E12
E1	0.47	0.06	0.00	0.06	0.06	0.12	0.00	0.06	0.00	0.06	0.00	0.12
E2	0.00	0.86	0.01	0.06	0.00	0.01	0.01	0.00	0.00	0.04	0.00	0.00
E3	0.02	0.00	0.91	0.00	0.02	0.00	0.00	0.02	0.00	0.00	0.02	0.00
E4	0.00	0.04	0.00	0.67	0.00	0.08	0.04	0.08	0.04	0.00	0.00	0.04
E5	0.01	0.00	0.02	0.00	0.89	0.00	0.00	0.01	0.00	0.02	0.04	0.00
E6	0.00	0.00	0.01	0.01	0.00	0.92	0.00	0.03	0.00	0.00	0.01	0.01
E7	0.00	0.00	0.02	0.03	0.00	0.00	0.91	0.02	0.02	0.00	0.00	0.02
E8	0.04	0.00	0.00	0.04	0.00	0.00	0.04	0.83	0.04	0.00	0.00	0.00
E9	0.01	0.01	0.03	0.00	0.02	0.01	0.01	0.01	0.92	0.00	0.00	0.00
E10	0.01	0.01	0.00	0.01	0.01	0.01	0.01	0.00	0.00	0.99	0.00	0.00
E11	0.01	0.02	0.00	0.00	0.00	0.01	0.00	0.00	0.02	0.00	0.93	0.02
E12	0.00	0.00	0.00	0.00	0.00	0.00	0.01	0.01	0.00	0.00	0.00	0.98
Accuracy						**91%**						

**TABLE 6:** Confusion matrix for correctness classification for UTKinect-Action3D dataset.

Class	Walk	Sit down	Stand up	Pick up	Carry	Throw	Push	Pull	Wave hands	Clap hands
Walk	0.90	0.00	0.20	0.00	0.00	0.00	0.01	0.05	0.01	0.00
Sit down	0.02	0.89	0.00	0.00	0.03	0.00	0.01	0.00	0.01	0.00
Stand up	0.02	0.01	0.60	0.01	0.03	0.00	0.03	0.05	0.00	0.00
Pick up	0.00	0.01	0.10	0.97	0.05	0.02	0.01	0.00	0.00	0.00
Carry	0.00	0.01	0.00	0.00	0.84	0.00	0.02	0.09	0.00	0.01
Throw	0.00	0.01	0.00	0.00	0.00	0.91	0.00	0.00	0.00	0.00
Push	0.05	0.01	0.00	0.00	0.03	0.02	0.92	0.00	0.00	0.01
Pull	0.00	0.03	0.10	0.00	0.00	0.00	0.00	0.82	0.01	0.01
Wave hands	0.00	0.01	0.00	0.01	0.03	0.05	0.01	0.00	0.97	0.00
Clap Hands	0.00	0.02	0.00	0.00	0.00	0.00	0.00	0.00	0.01	0.98
Accuracy						**94.2**%				

### 4.4 Classification performance evaluation

Table 7 reports the precision, recall, and F1-score values for correctness classification on the KIMORE dataset. For the KIMORE dataset, the model demonstrated excellent performance across all five exercise classes. The highest scores were achieved for Exercise 5 (E5), with a precision of 0.98, recall of 0.96, and an F1-score of 0.97, followed by Exercise 4 (E4), which recorded consistent values of 0.96 for both precision and recall, resulting in an F1-score of 0.96. While Exercise 1 (E1) also achieved strong results with a precision of 0.93 and recall of 0.95, slightly lower values were observed for Exercise 2 (E2) and Exercise 3 (E3), with F1-scores of 0.89 and 0.87, respectively. These lower values correspond with the confusion matrix findings, where misclassifications between E2 and E3 were noted, highlighting areas where the system occasionally struggles to differentiate similar movement patterns.

**TABLE 7:** Precision, recall, and F1-score results over KIMORE Dataset.

Exercises	Precision	Recall	F1-score
E1	0.93	0.95	0.94
E2	0.89	0.90	0.89
E3	0.86	0.87	0.87
E4	0.96	0.96	0.96
E5	0.98	0.96	0.97

Table 8 reports the precision, recall, and F1-score values for the mRI dataset across twelve different exercise classes. The model demonstrated strong and consistent performance on most exercises. Notably, E11 achieved the highest scores with a precision of 0.93, recall of 0.92, and F1-score of 0.93, followed closely by E10 (precision: 0.89, recall: 0.94, F1-score: 0.92) and E3 (precision: 0.91, recall: 0.92, F1-score: 0.91). Exercises E5, E7, E9, and E12 also showed high F1-scores of 0.89, indicating robust classification in these categories. However, E1 exhibited the lowest performance, with a recall of 0.47 and an F1-score of 0.59, suggesting challenges in accurately identifying this exercise. Overall, the model shows promising recognition capability across the dataset, with a few classes like E1 and E4 (F1-score: 0.72) requiring further attention to enhance classification accuracy.

**TABLE 8:** Precision, recall, and F1-score results over mRI dataset.

Exercises	Precision	Recall	F1-score
E1	0.82	0.47	0.59
E2	0.86	0.87	0.86
E3	0.91	0.92	0.91
E4	0.76	0.68	0.72
E5	0.89	0.90	0.89
E6	0.79	0.93	0.86
E7	0.88	0.89	0.89
E8	0.78	0.84	0.81
E9	0.88	0.90	0.89
E10	0.89	0.94	0.92
E11	0.93	0.92	0.93
E12	0.82	0.98	0.89

Table 9 reports the precision, recall, and F1-score values for the UTKinect-Action3D dataset. The model performed well across all actions, with Clap hands achieving the highest scores (precision: 0.97, recall: 0.97, F1-score: 0.97), followed by Throw (precision: 0.91, recall: 0.99, F1-score: 0.95) and Pick up (precision: 0.98, recall: 0.84, F1-score: 0.90). Other actions like Carry and Push also showed strong results. However, Stand-up had lower performance (precision: 0.60, recall: 0.80, F1-score: 0.69), indicating difficulties in differentiation. Overall, the model demonstrated strong action recognition, with room for improvement in Stand-up classification.

**TABLE 9:** Precision, recall, and F1-score results over UTKinect-Action3D dataset.

Exercises	Precision	Recall	F1-score
Walk	0.91	0.77	0.83
Sit down	0.89	0.93	0.91
Stand up	0.60	0.80	0.69
Pick up	0.98	0.84	0.90
Carry	0.83	0.87	0.85
Throw	0.91	0.99	0.95
Push	0.91	0.88	0.90
Pull	0.81	0.85	0.83
Wave hands	0.96	0.90	0.93
Clap hands	0.97	0.97	0.97

**FIGURE 12:**
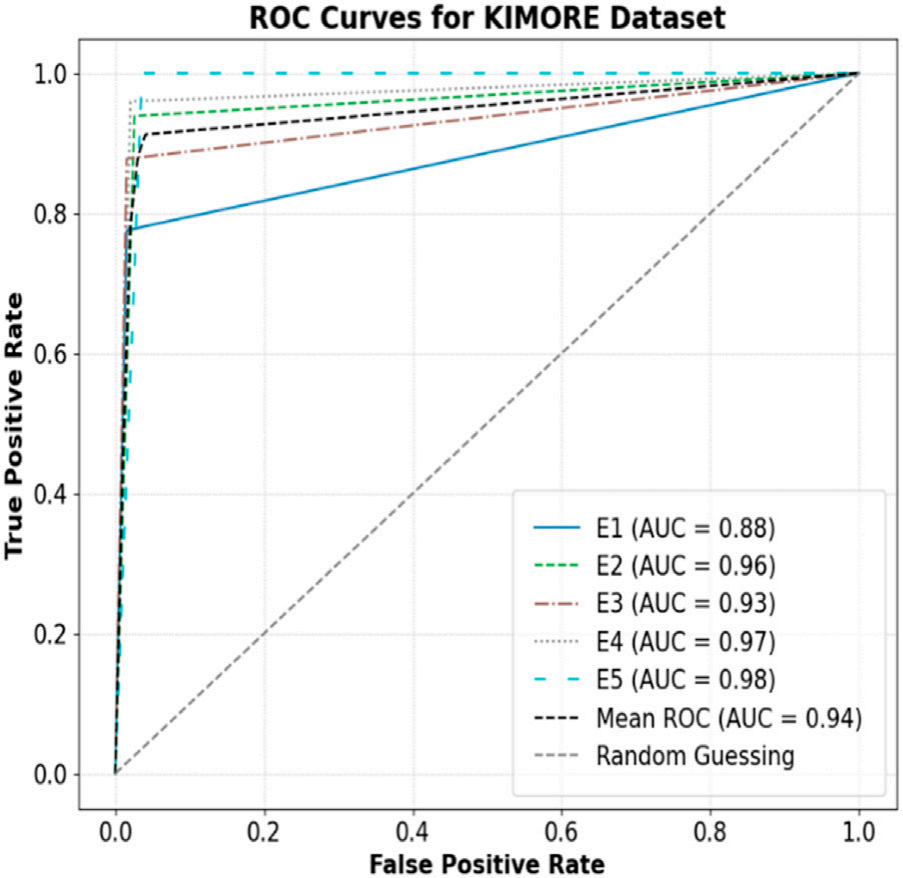
ROC Curves for KIMORE dataset.

**FIGURE 13:**
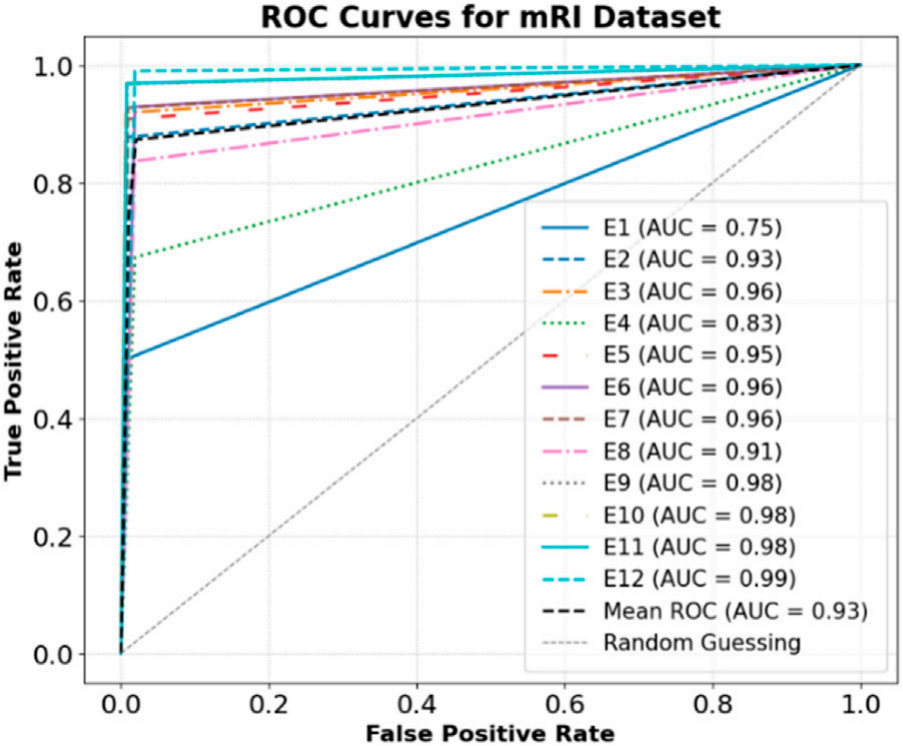
ROC Curves for mRI dataset.

The model showed strong classification on the KIMORE dataset (Figure 12), with high AUCs across exercises. E5 (0.98), E4 (0.97), and E2 (0.96) had near-perfect discrimination, while E1, though lower, still achieved 0.88. The mean AUC was 0.94, highlighting robust overall performance, with E1 likely being harder to distinguish due to movement similarities. The model showed strong discriminative performance across all exercises (E1–E12) in the mRI dataset, as shown by the ROC analysis in Figure 13. Most exercises achieved excellent AUCs, with E12 (0.99), E9–E11 (0.98), and E3, E6, and E7 (0.96) performing exceptionally well. E2 (0.93) and E8 (0.91) also maintained high performance. The mean AUC was 0.93, far above random guessing. E1 (0.75) and E4 (0.83) had lower scores, suggesting greater classification challenges due to overlapping kinematics.

**FIGURE 14:**
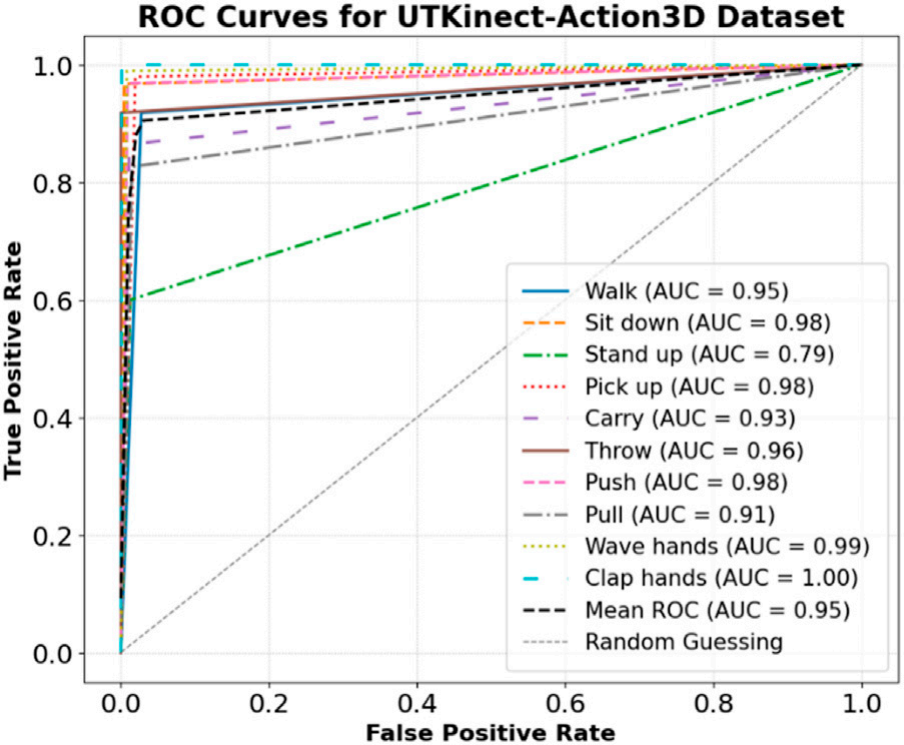
ROC Curves for UTKinect-Action3D dataset.

Overall, the model demonstrated robust exercise recognition in the mRI dataset. The model showed strong performance on the UTKinect-Action3D dataset (Figure 14), with most actions achieving high AUCs. “Clap hands” reached 1.00, “Wave hands” 0.99, and “Sit down,” “Pick up,” and “Push” all 0.98. “Stand up” had a lower AUC of 0.79. The mean AUC was 0.95, indicating robust classification with only minor challenges for “Stand up.”

### 4.5 Comparison with state-of-the-art

Table 10 compares recent studies on rehabilitation exercise recognition. Jleli et al. (2024) achieved 87% accuracy with YOLO V5 and ShuffleNet V2 on KIMORE, while Zaher et al. (2024) improved it to 93.08% with CNN optimization. Zaher et al. (2025) reported 81.85% using a hybrid FCBF-Extra Trees model. For UTKinect-Action3D, Keçeli et al. (2022), Ding et al. (2018), and Kumar et al. (2024) achieved 93.4%, 91.5%, and 93.5% accuracy, respectively. An et al. (2022) achieved mAP scores of 91.56% and 95.07% with ActionFormer on mRI. The proposed model outperforms previous work with 94.73% (KIMORE), 91% (mRI), and 94.2% (UTKinect-Action3D), demonstrating superior generalizability.

**TABLE 10:** Comparison of methodologies, datasets, and results from recent studies on physical rehabilitation exercise recognition and assessment.

Author	Title	Methodology	Dataset	Results
Jleli et al. (2024)	Artificial Intelligence-driven Remote Monitoring Model for Physical Rehabilitation	YOLO V5–ShuffleNet V2	KIMORE	Accuracy = 87.00%
Zaher et al. (2024)	Unlocking the potential of RNN and CNN models for accurate rehabilitation exercise classification on multi-datasets	CNN with hyperparameter tuning	KIMORE	Accuracy = 93.08%
Zaher et al. (2025)	Rehabilitation monitoring and assessment: a comparative analysis of feature engineering and machine learning algorithms on the UI-PRMD and KIMORE benchmark datasets	The combination of FCBF for feature ranking and Extra Trees classifier	KIMORE	Accuracy = 81.85%
Keçeli et al. (2022)	3D Skeletal Volume Templates for Deep Learning-Based Activity Recognition	HOG + Deep Features	UTKinect-Action3D Dataset	Accuracy = 93.40%
Ding et al. (2018)	Human Action Recognition Using Similarity Degree Between Postures and Spectral Learning	Rotation Matrix Representation-Based 3D (RMRB3D) with Singular Value Decomposition (SVD) and Hidden Markov Model (HMM)	UTKinect-Action3D Dataset	Accuracy = 91.50%
Kumar et al. (2024)	Human Action Recognition from Depth Sensor via Skeletal Joint and Shape Trajectories with a Time-Series Graph Matching	Time-Series Graph Matching (TSGM)	UTKinect-Action3D Dataset	Accuracy = 93.50%
An et al. (2022)	Multi-modal 3D Human Pose Estimation using mmWave, RGB-D, and Inertial Sensors	ActionFormer	mRI: Multi-modal 3D Human Pose Estimation Dataset using mmWave, RGB-D, and Inertial Sensors	Protocol 1 (Random split) mAP = 91.56 Protocol 2 (Subject-wise split) mAP = 95.07
Proposed		KMORE	**94.73%**	
		mRI	**91.00%**	
		UTKinect-Action3D	**94.20%**	

### 4.6 Ablation study

An ablation study was performed to evaluate the contribution of each feature stream as well as the impact of feature dimensionality on model performance (Table 11). The full model, which integrates preprocessing, 3D Mesh features, 2D keypoints, and BPL-based contour points, achieved the highest accuracy across all datasets. For the 3D Mesh features, reducing the number of SMPL vertices by 25% and 50% produced only moderate accuracy declines compared to the full-resolution mesh, while complete removal caused the largest performance drop (KIMORE: 94.73%–91.79% to 90.52%–87.00%). The smooth decline across these conditions indicates that the model does not simply memorize high-dimensional details but continues to generalize well even with fewer vertices. This suggests a low risk of overfitting to mesh complexity, while still confirming the strong importance of biomechanical information. For the 2D keypoints, models trained with individual detectors (AKAZE, SIFT, BRISK, ORB, Shi-Tomasi) achieved stable accuracy in the 90%–93% range, while the fused vector consistently outperformed single detectors. This consistency across different detectors demonstrates that the model is not overfitting to the idiosyncrasies of any one keypoint representation. Instead, it learns complementary information from multiple detectors, thereby improving generalization and robustness.

**TABLE 11:** Ablation study on model configurations and their impact on exercise recognition accuracy across KIMORE, UTKinect-Action3D and mRI datasets.

Model configuration	Description	KIMORE accuracy (%)	mRI accuracy (%)	UTKinect-Action3D accuracy (%)
All Parameters (Preprocessing, 3D Mesh, 2D Keypoints, BPL-based Contour Points)	Model trained using all feature extraction techniques	94.73%	91.00%	94.20%
Without Preprocessing	Model trained without image preprocessing	90.50%	87.30%	88.80%
Without 25% 3D Mesh Vertices	Model trained with 25% reduced 3D Mesh Vertices	91.79%	87.50%	88.80%
Without 50% 3D Mesh Vertices	Model trained with 50% reduced 3D Mesh Vertices	90.52%	87.10%	86.20%
Without 3D Mesh	Model trained without 3D mesh features	87.00%	85.00%	82.00%
Without 2D Keypoint (AKAZE)	Model trained with AKAZE keypoints	91.20%	89.40%	92.25%
Without 2D Keypoint (SIFT)	Model trained with SIFT keypoints	90.25%	88.20%	90.20%
Without 2D Keypoint (BRISK)	Model trained with BRISK keypoints	91.50%	89.10%	91.90%
Without 2D Keypoint (ORB)	Model trained with ORB keypoints	90.75%	89.40%	91.80%
Without 2D Keypoint (Shi Tomasi)	Model trained with Shi Tomasi keypoints	92.30%	89.70%	93.20%
Without Complete 2D Keypoints Vector	Model trained without all 2D keypoint features	91.00%	89.00%	86.00%
Without 50% BPL-based Contour Points	Model trained without 50% BPL-based contour points (Random Selection with Uniform number of keypoints per body part)	92.70%	90.40%	91.20%
Without BPL-based Contour Points	Model trained without BPL-based contour points	91.60%	89.40%	88.90%

For the BPL-based contour features, randomly pruning 50% of contour points while maintaining uniform distribution across body parts led to only a minor accuracy drop (KIMORE: 94.73%–92.70%), while complete removal produced a slightly larger decline. This indicates that the framework remains reliable even when partial information is missing, showing resilience to noise and occlusion. Overall, the ablation results confirm that the model maintains strong performance under reduced feature dimensionality and noisy conditions, demonstrating both robustness and resistance to overfitting.

### 4.7 Computational cost analysis

We evaluated the computational cost of all the major components of proposed architecture as shown in Table 12, the pipeline exhibits a clear distinction between lightweight classical techniques and computationally intensive deep learning models. Classical keypoint detection methods, including corner and feature detection, are highly efficient and contribute minimally to overall computational cost. In contrast, stages such as semantic segmentation, body part parsing, 3D keypoint extraction, and pose fitting dominate processing, forming the primary bottlenecks in the system. Feature fusion operations are lightweight, while transformer-based inference introduces moderate computational overhead. Overall, the pipeline relies on GPU acceleration for near real-time performance, with optimization of segmentation and pose-fitting stages offering the greatest potential for improving throughput.

**TABLE 12:** FLOPs, and estimated time per frame for 3D-PoseFormer pipeline.

Stage	Technique	Estimated time per frame (ms)	FLOPs (GFLOPs)
RGB-KPD	Shi-Tomasi Corner Detection	1.2	0.02
	AKAZE Feature Detection	1.3	0.03
	BRISK Feature Detection	1.3	0.03
	SIFT Feature Detection	1.8	0.04
	Harris Corner Detection	1.2	0.02
	DeepLabV3+ with ResNet-101 (Segmentation)	12.0	15.6
	Body Part Labeling (Single-Human-Parsing-LIP)	2.5	0.8
	Contour-Based Keypoint Extraction	0.6	0.01
D-Mesh	DKP-Net-24 (3D Keypoint Extraction, L and R pipelines)	12.0	0.5
	SMPL Fitting (Pose/Shape Optimization)	18.0	2.3
Feature Fusion	Concatenation of RGB and Depth Features	2.5	0.01
Transformer Inference	Transformer Encoder (4 layers, 8 heads, 512 dims)	10.0	1.8
Total		∼55	20.31

## 5 Conclusion

In this work, we proposed a novel multimodal deep learning pipeline for automated recognition and assessment of physiotherapy exercises, specifically designed for remote rehabilitation of physically disabled individuals. Unlike existing systems that rely on wearable sensors, markers, or controlled clinical environments, our framework leverages only RGB and depth data to deliver accurate, real-time performance evaluation in unconstrained, home-based settings. The key novelty of the proposed approach lies in its comprehensive fusion of depth-based 3D body mesh representations generated using SMPL and appearance-based features extracted from RGB images using both classical keypoint detectors and semantic contour analysis on segmented body parts. This multi-level feature representation is further enhanced by a Transformer-based temporal modeling module, enabling robust classification and fine-grained assessment of exercise execution quality. Our system outperforms prior methods on benchmark datasets, achieving 94.73% accuracy on KIMORE, 91% on mRI and 94.2% on UTKinect-Action3D demonstrating its effectiveness, generalizability, and real-world applicability. The proposed pipeline represents a significant advancement toward intelligent, scalable, and sensor-free telerehabilitation solutions.

## Data Availability

Publicly available datasets were analyzed in this study. This data can be found here: https://vrai.dii.univpm.it/content/kimore-dataset; UTKinect-Action3D Dataset: https://cvrc.ece.utexas.edu/KinectDatasets/HOJ3D.html; mRI: Multi-modal 3D Human Pose Estimation Dataset using mmWave, RGB-D, and Inertial Sensors: https://sizhean.github.io/mri.
